# Safety and efficacy of the mesenchymal stem cell in feline eosinophilic keratitis treatment

**DOI:** 10.1186/s12917-018-1413-4

**Published:** 2018-03-27

**Authors:** Antonio J. Villatoro, Silvia Claros, Viviana Fernández, Cristina Alcoholado, Fernando Fariñas, Antonio Moreno, José Becerra, José A. Andrades

**Affiliations:** 1 ImmuneStem, Instituto de Inmunología Clínica y Terapia Celular, 29018 Málaga, Spain; 2 Laboratory of Bioengineering and Tissue Regeneration (LABRET), Department of Cell Biology, Genetics and Physiology, Faculty of Sciences, University of Málaga, Biomedicine Research Institute of Malaga (IBIMA), Campus Universitario de Teatinos, 29071 Málaga, Spain; 3 Networking Biomedical Research Center in Bioengineering, Biomaterials and Nanomedicine (CIBER-BBN), 28029 Madrid, Spain; 4 Hospital veterinario Alhaurín el Grande. Alhaurín el Grande, 29120 Málaga, Spain; 5 Laboratory of Bioengineering and Tissue Regeneration, Andalusian Center for Nanomedicine and Biotechnology-BIONAND, 29590 Málaga, Spain

**Keywords:** Cat, Adipose mesenchymal stem cell, Lacrimal gland, Feline eosinophilic keratitis, Feline herpes virus, Allogeneic cell therapy

## Abstract

**Background:**

Feline eosinophilic keratitis (FEK) is a chronic keratopathy caused by a suspected immune mediated response to an unknown antigenic stimulus. The purpose of this study was to investigate the safety and therapeutic effects of allogeneic feline adipose-derived mesenchymal stromal cells (fAd-MSCs) implanted subconjunctival around the ocular surface lesion in five cats with FEK refractory to current available treatments.

**Results:**

FEK was diagnosed by clinical appearance and evidence of eosinophil and/or mast cells in corneal cytology. Each animal was treated with two applications of 2 × 10^6^ million of fAd-MSCs 2 months apart. Ocular surface integrity was assessed before treatment and at 1, 3, 6 and 11 months after treatment. Clinical signs showed a significant change during the follow-up with resolution of the corneal and conjunctiva lesions and there were no signs of regression or worsening.

**Conclusions:**

Implanted cells were well-tolerated and effective reducing clinical signs of FEK with a sustained effect during the study period. None of the animals showed systemic or local complications during the study. To our knowledge, this is the first time in literature that local implantation of allogeneic fAd-MSCs has been found as an effective therapeutic alternative to treat cats with FEK.

## Background

Feline eosinophilic keratitis (FEK) or feline proliferative keratitis (FPK) is a unique, chronic and progressive, infiltrative keratopathy in cats [1, 2], but is also described in horses [3], rabbits [4], and dogs [5]. This disease involves the ocular surface in cats of any age, but is most commonly diagnosed in young to middle-aged cats, and appears unilaterally 66% of the time, but eventually become a bilateral condition [1, 2, 6]. In general, the condition only affects the cornea, though it can also involve the third eyelid and the conjunctiva [1].

The diagnosis is based on clinical appearance and corneal cytology. Clinical signs start with a superficial vascularization of the perilimbal corneal, and as the disease progresses, the lesions appear as an edematous area, an irregular and vascularized mass with pink to white infiltrates that form gritty yellow-white corneal plaques extending axially from the limbus.

The occurrence of eosinophil and/or mast cells in a corneal cytologic specimen is diagnostic for proliferative feline eosinophilic keratitis [1, 2, 7]. The etiology of this condition is not clearly understood. FEK is believed to be an immune-mediated disorder in which there is an exaggerated immune response to an antigenic stimulus, with a type I or type IV (subtype IVb) hypersensitivity reaction [8].

There may be an underlying viral infection, feline herpes virus-1 (FHV-1) that plays an initial role in the pathogenesis of the disease [2, 6, 9, 10], having been described that at least 76.3% of cats with FEK are positive to FHV-1 [6].

The current treatment of FEK typically consists of topical applications of corticosteroids or similar and immunosuppressive drugs as cyclosporine A, several times a day for long periods of time with variability in efficacy and safety [1, 2, 6, 11]. In addition, recurrences of clinical signs are common after cessation of treatment [6, 10, 11].

Adipose-derived mesenchymal stromal cells (Ad-MSCs) are multipotent stem cells with capacity to differentiate into osteogenic, adipogenic, chondrogenic, myogenic, among other cell lineages, and with important secretory abilities of different bioactive molecules with trophic, paracrine and immunomodulatory functions [12–16]. Although the mechanisms of immunomodulation remain partially elusive, recent studies have demonstrated the capacity of MSCs to modulate both the innate and adaptive immune systems [13, 17]. MSCs inhibit T-cell proliferation, alter B-cell function, downregulate major histocompatibility complex (MHC) II, and inhibit dendritic cell maturation and differentiation [13, 18, 19].

Their low immunogenicity and their immunoregulatory potential allow their allogeneic use, which makes them an alternative to be a promising new treatment for severe refractory autoimmune diseases [16, 20]. They have been extensively studied as a cellular therapy for different pathological conditions, with the cat and dog as animal models [21–24].

The purpose of our study was to evaluate the safety and the therapeutic effects of local implantation of allogeneic feline Ad-MSCs (fAd-MSCs) subconjunctivally in cats with an 11-month follow-up. fAd-MSCs were previously characterized through in vitro tests, according to the International Society of Cell Therapy.

## Methods

This was an uncontrolled open-label study in the treatment of FEK.

All animal procedures were conducted by licensed veterinary surgeons and comply with both national and European legislation (Spanish Royal Decree RD1201/2005 and EU Directive 86/609/CEE as modified by 2003/65/CE, respectively) for the protection of animals used for research experimentation and other scientific purposes. Likewise, the protocols were approved by the Institutional Animal Care and Use Committee of BIONAND (Andalusian Center for Nanomedicine and Biotechnology) Málaga, Spain, and writing consent was obtained from all owners.

### Animals

Five client-owned cats (5 eyes) of European breeds, 3 males and 2 females, aged between 3 and 6 years, and weighting from 3.2 to 4 kg were selected (Table 1).

**Table 1:** Summary of signalment, clinical data and evolution for veterinary patients

Parameter	Cat-1	Cat-2	Cat-3	Cat-4	Cat-5
Age (years)	4	4	5	6	3
Sex	MC	MC	MC	FS	FS
Breed	DSH	DSH	DSH	DSH	DSH
Weight (kg)	3.2	3.8	3.8	4	4.1
Duration (months)	12	9	8	10	6
Eye	R	L	L	R	R
Lesion localization	ST	ST	IN	IN	ST
FHV-1	–	+	+	+	+
Clinical signs	PL,V, H	PL, V, ED, H	PL, V, ED, H	PL, V, H	PL, V, H
STT	14	18	16	21	19
Cytology	E, M	E, M	E, M, N	E, M	E, M
Previous treatment	C, Ca, Ao	Ca, Ly, Ao	Ca, C, Ly, Ao	Ca, C, Ly, IΩ, Ao	Ca, Ly, IΩ, Mg, Ao
Clinical remission	2 months	2 months	6 months	4 months	5 months
Hematologic changes	No	No	No	No	No

MC, male castrated; FS, female spayed; DSH, domestic short hair; R, right eye; L, left eye; ST, superotemporal quadrant; IN, inferonasal quadrant; FHV-1, feline herpes virus 1; PL, corneal infiltrative plaque; V, vascularization; H, hyperemia; ED, corneal edema; STT, Schirmer tear test; E, eosinophils; M, mast cells; N, neutrophils; C, topical corticosteroids; Ca, topical cyclosporine A; Ao, antibiotics; Ly, L-lysine; IΩ, topical interferon omega; Mg, oral megestrol acetate

All individuals were affected by FEK, at least during 6 months and refractory to the current treatment, with duration of the clinical signs from 6 to 12 months (mean 9 months). Only one eye was affected in all cases. The right eye was affected in 3 cats (60%), the left eye in 2 cats (40%). They received previous treatment (corticosteroids, cyclosporine, and others) with partial improvement and recurrences with some level of intolerance to cyclosporine administration without any viable therapeutic alternatives (Table 1). Untreated or placebo animals were not included in the present study, since only cats with naturally occurring FEK were admitted, and spontaneous recovery has never been reported in cats with refractory FEK [6].

Cats did not receive any kind of anti-inflammatory or immunomodulatory medications for at least 2 weeks before cell therapy treatment, and for the entire duration of the study. All cat owners signed a written consent before initiation of this experimental procedure and were fully informed that long-term outcome, safety, complications, and efficacy of the cell implantation in FEK were not known.

### Clinical evaluation

All animals underwent a full veterinarian clinical and ophthalmic examination.

Initial complete ophthalmic examination consisted of Schirmer tear test (STT-1) at one minute, fluorescein staining, applanation tonometry, slit-lamp biomicroscopy and indirect ophthalmoscopy. The corneal and conjunctival lesions were described in each animal, affected eye and position of the ocular surface lesion (Table 1).

Diagnosis of FEK was based upon the clinical appearance of infiltrates in one of the eyes and the corneal and conjunctival cytology.

Clinical findings: Corneal vascularization and infiltration was present in all cases. Proliferative, white-pink, edematous, irregular and vascularized ingrowth of tissue and gritty, white-yellow corneal plaques were prominent in all cases, spreading from the limbus to the center of the cornea. Corneal lesions were found in the superotemporal quadrant in 3 of the eyes (60%), and into the inferior nasal quadrant in two eyes (40%). None of the cases showed corneal ulcer and the fluorescein staining was negative. The adjacent conjunctiva was hyperemic near to the corneal lesion in all cases. The ocular alteration was limited to the cornea and conjunctiva, and all the others ophthalmologic parameters evaluated were normal.

**Fig. 1:**
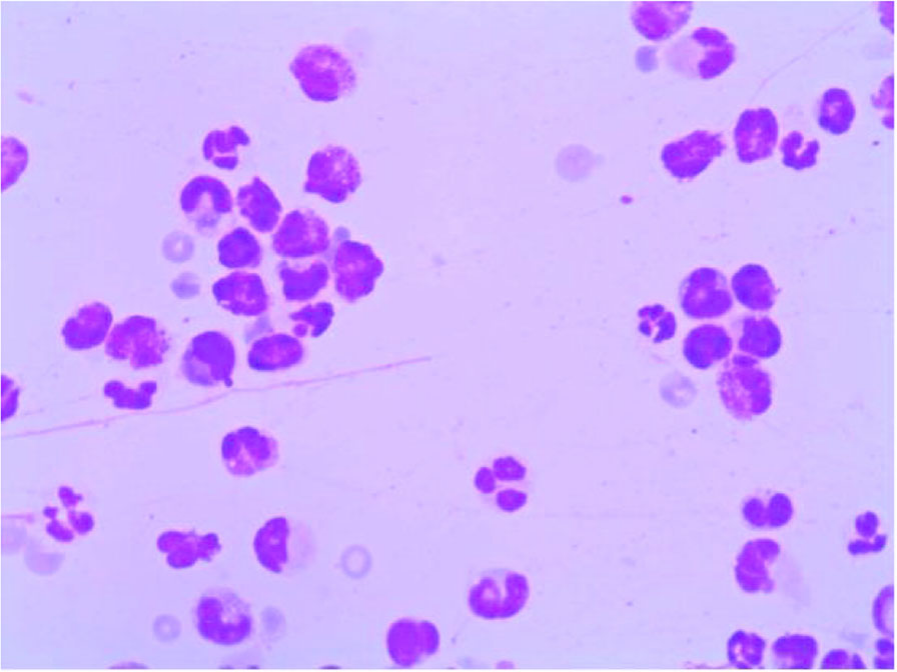
Corneal cytology. Abundant eosinophils and mast cells were detected in all animals

Cytology was obtained in all cases after application of a topical anesthetic with the blunt end of a scalpel blade. Abundant eosinophils and mast cells were detected in all animals (Fig. 1). Besides, one of the cats showed the existence of a remarkable number of neutrophils. The Wright-Giemsa stains were negative to microorganisms.

A polymerase chain reaction (PCR) for FHV-1 was performed by Laboratory Idexx (Barcelona, Spain), applying a hydrophilic polyethersulfone membrane on the cornea. FHV-1 was detected in four cats of this study.

A complete blood cell count and serum biochemistry profile prior to treatment were performed on all animals, without showing any alteration of the parameters evaluated. Acute phase markers were not included in the study, as no specific parameter is described for this disease.

**Fig. 2:**
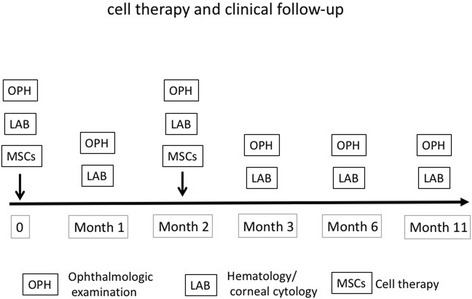
Summary of the cell therapy and clinical follow-up

Follow up was at 1, 2, 3, 6 and 11 months after fAd-MSCs implantation (Fig. 2). During this time, recurrences of the corneal lesion, additional ocular diseases, and the health of the contralateral cornea were monitored. Corneal cytology, complete blood cell count and serum biochemistry profile were performed during all follow up appointments.

### Isolation and in vitro culture of fAd-MSCs

Adipose tissue was aseptically collected from abdominal subcutaneous fat under general anesthesia with isoflurane of a single specific pathogen-free cat during a routine ovariectomy procedure, and maintained at 4 °C in a tube with culture medium. Under a laminar flow hood, after weighing the harvested adipose tissue, 5 g of fat was minced and mixed with 20 mL of Hanks solution (Sigma-Aldrich, Madrid, Spain) containing 0.1% collagenase type II (Sigma-Aldrich) by incubating at 37 °C for 90 min in orbital agitation. After digestion, the cell suspension was filtered through a 100 μm cell strainer. The cell suspension was centrifuged at 400 g for 5 min to discard the lipid layer and the obtained cell pellet was washed with culture medium. Primary cultures were carried out in T175 flasks with Dulbecco’s modified Eagle’s medium (DMEM) containing 10% (*v*/v) fetal bovine serum (FBS), 2.5 mM L-glutamine, 100 U/mL penicillin, 100 μg/mL streptomycin, and 1.25 μg/mL fungizone (all from Sigma-Aldrich). The culture medium was changed twice per week and cells were selected by their capacity to attach to the flask surface, discarding the floating cells in the first medium change at 72 h. When culture flasks became 80% semiconfluence, cells were detached with 0.25% trypsin containing 1 mmol/L EDTA and subsequently replated at a concentration of 10^4^ cells/cm^2^ for continued passaging. The remaining cells were cryopreserved in cryopreservation media (10% dimethyl sulfoxide and 90% FBS), frozen at − 80 °C in an isopropanol jacketed closed container (Nalgene Cryo freezing container), and stored in liquid nitrogen until the next day. All experiments and in vivo implantation were conducted at passage 2.

### Cell proliferation

Cell proliferation was measured using MTS (CellTiter 96 Aqueous One Solution Cell Proliferation Assay, Promega) assay according to manufacturer protocol. Briefly, in a 96-well plate, 3000 fAd-MSCs per well were seeded using 8 wells as replicates of each sample. Cells were allowed to proliferate performing the medium change twice per week and making readings on days 0, 3, 5, 7 11, 14, 18, 21, 25 and 28. Supernatants were collected and absorbance was measured at 490 nm using a microplate reader (ELx800, Bio-Tek instruments, Winooski, VT, USA).

### Flow cytometry analysis

Fluorescence-activated cell sorting (FACS) was used to characterize fAd-MSCs from passage 2, and was performed as previously described in detail [25]. The antibodies used are listed in Table 2.

**Table 2:** Antibodies used for flow cytometry

Antibody	Supplier	Clone	Isotype	Fluorochrome
CD29	Miltenyi Biotech	TS2/16	IgG1 κ	PE
CD34	Miltenyi Biotech	AC136	IgG2a	FITC
CD44	Miltenyi Biotech	DB105	IgG1	APC
CD45	Miltenyi Biotech	5B1	IgG2a	APC
CD73	BD Pharmingen	AD2	IgG1 κ	PE
CD90	Miltenyi Biotech	DG3	IgG1	APC
MHC-I	BD Pharmingen	G46–2.6	IgG1 κ	FITC
MHC-II	BD Pharmingen	G46–6	IgG2a κ	PE
STRO-1	R&D Systems	STRO-1	IgM λ	Pure
Anti-mouse IgM	AbD Serotec	3A6	Polyclonal	PE

### In vitro multilineage cell differentiation

To assess the multipotentiality, fAd-MSCs at passage 2 were differentiated long adipogenic, osteogenic, and chondrogenic lineages according to standard protocols, as previously reported [26, 27]. Cells were cultured in specific induction medium for 21 days. Afterward, adipogenic differentiation was evaluated by Oil red O staining on day 21 after induction. Alkaline phosphatase (ALP) activity and calcium deposition were analyzed on days 7, 14 and 21 to evaluate osteogenic differentiation. For chondrogenic differentiation, a 3D pellet culture model was used. On day 21, pellets were subjected to routine histological processing and then stained by toluidine blue (TB), alcian blue (AB), and immunohistochemically for type II collagen, using standard techniques as previously described [24].

### Karyotype

fAd-MSCs at passage 2 were karyotyped as previously described [28]. fAd-MSCs were seeded and cultured in a T175 flask until semi-confluence was achieved. Then, cells were harvested and treated with 0.05 μg/ml of colcemid (Thermo Fisher Scientific, Inc.) for 20 min to arrest mitotic cells in metaphase. Subsequently, pelleted cells were resuspended in a hypotonic solution (0.075 M potassium chloride solution) for 5 min to swell cells. fAd-MSCs were then fixed in cold methanol: glacial acetic acid (3:1) and washed three times to ensure complete removal of cytoplasmic debris. Afterward, they were stained in 2% Giemsa and analyzed with ordinary bright-field microscopy. Analysis included scanning all slides, counting a minimum of twenty metaphases, analyzing a minimum of seven metaphases, and karyotyping a minimum of two metaphases.

### Immunomodulatory potential

Inhibition of lymphocyte proliferation assays were performed using peripheral blood mononuclear cells (PBMCs) harvested from a healthy donor cat. PBMCs were separated using Ficoll-Hypaque density gradient centrifugation and stained with 4 μM 5-chloromethylfluorescein diacetate (CMFDA, Cell Tracker Green Kit C2925, Thermo Fisher Scientific, Inc.). PBMCs were plated in a 96-well plate at a concentration of 5 × 104 cells/well. fAd-MSCs at passage 2 were harvested and seeded in the 96-well plate at 1 × 104 cells/well, previously inactivated with mitomycin C for 3 h. Concanavalin A (ConA; Sigma- Aldrich) was added to experimental wells at a final concentration of 5 μg/ml. The following experimental groups were used (in triplicate): PBMCs control; PBMCs and fAd-MSCs; PBMCs and ConA; PBMCs, fAd-MSCs and ConA. Cells were incubated for 72 h at 37 °C and 5% CO2, and then, the amount of lymphocyte proliferation was analyzed by flow cytometer (Beckman Coulter). For the purpose of comparison, lymphocytes stimulated with ConA were set to 100% proliferation. Flow cytometry data were analized using FlowJo cytometry software.

### Cell transplantation

The procedure was performed under sedation with metomidine 0.005 mg/Kg (Sedator® Lab. DVF, Barcelona, Spain).

All eyes were implanted aseptically, with two injections in a time interval of 2 months, of 2 × 10^6^ allogeneic fAd-MSCs in 0.4 mL DMEM, using a 21G needle, subconjunctivally near the lesion, with preliminary vitality test with trypan blue staining. All cats were hospitalized for 24 h after local transplantation to monitor any potential adverse effect.

The animals did not receive any kind of systemic and topical medication during the follow up period.

### Statistical analysis

Means and standard deviations were performed using Sigma Stat software (SPSS Inc., Chicago, IL) with Student’s t tests or one-way analysis of variance (ANOVA) after the data passed normality and equal variance tests. Results were considered significantly different at *p* < 0.05.

## Results

### Isolation and culture of fAd-MSCs

**Fig. 3:**
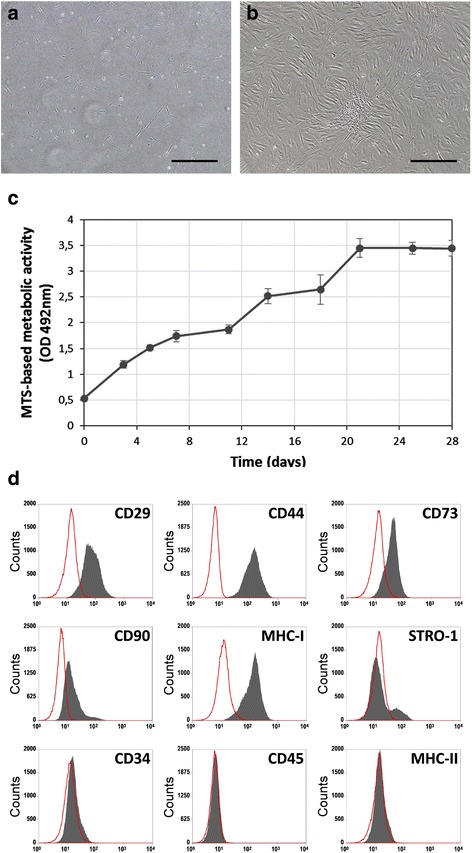
Cell morphology, proliferation and representative FACS analysis of fAd-MSCs for several mesenchymal and hematopoietic markers. **a** In primary cultures, a large number of adherent cells with fibroblastic morphology and colony-forming units were observed two days after initial seeding. **b** On secondary cultures, fAd-MSCs appeared as spindle-shaped cells that grown in a monolayer. **c** Representative curve obtained with MTS cell proliferation assay at passage 2. Cells started proliferating immediately after being plated, initiating the logarithmic growth phase and reaching its plateau phase around 21 days. **d** The immunophenotype profiles revealed a homogeneous cell population, characterized by the strong positive expression of CD29, CD44, CD73, CD90 and major histocompatibility class I (MHC-I), and lack expression of CD34, CD45 and MHC-II. As well, a minor population of STRO-1-expressing cells was observed. Bars, 200 μm

The abdominal subcutaneous fat was processed and MSCs were successfully isolated from the donor sample using their ability to adhere to tissue culture plastic. In primary cultures, the cells grew rapidly and a large number of colony-forming units was observed 2 days after initial seeding and 80% of semiconfluence was achieved on day 14 (Fig. 3a). On secondary cultures, fAd-MSCs appeared as spindle-shaped cells that were grown in a monolayer and maintained their proliferation capacity without indication of senescence (Fig. 3b).

### Cell proliferation

Cell proliferation was studied by MTS assay. The growth curve showed that the cells started proliferating immediately after being plated (there was no lag phase), initiating the logarithmic growth phase and reaching its plateau phase in approximately 21 days (Fig. 3c).

### Flow cytometry analysis

Once the secondary cultures at passage 2 reached 70% of confluence, cells were subjected to FACS analysis. The profiles of fAd-MSCs were uniformly and strongly positive for mesenchymal markers CD29, CD44, CD73, CD90 and MHC-I (Fig. 3d), and negative for hematopoietic markers CD34, CD45 and MHC-II. Additionally, a minor population of STRO-1-expressing cells was observed.

### In vitro multilineage cell differentiation

**Fig. 4:**
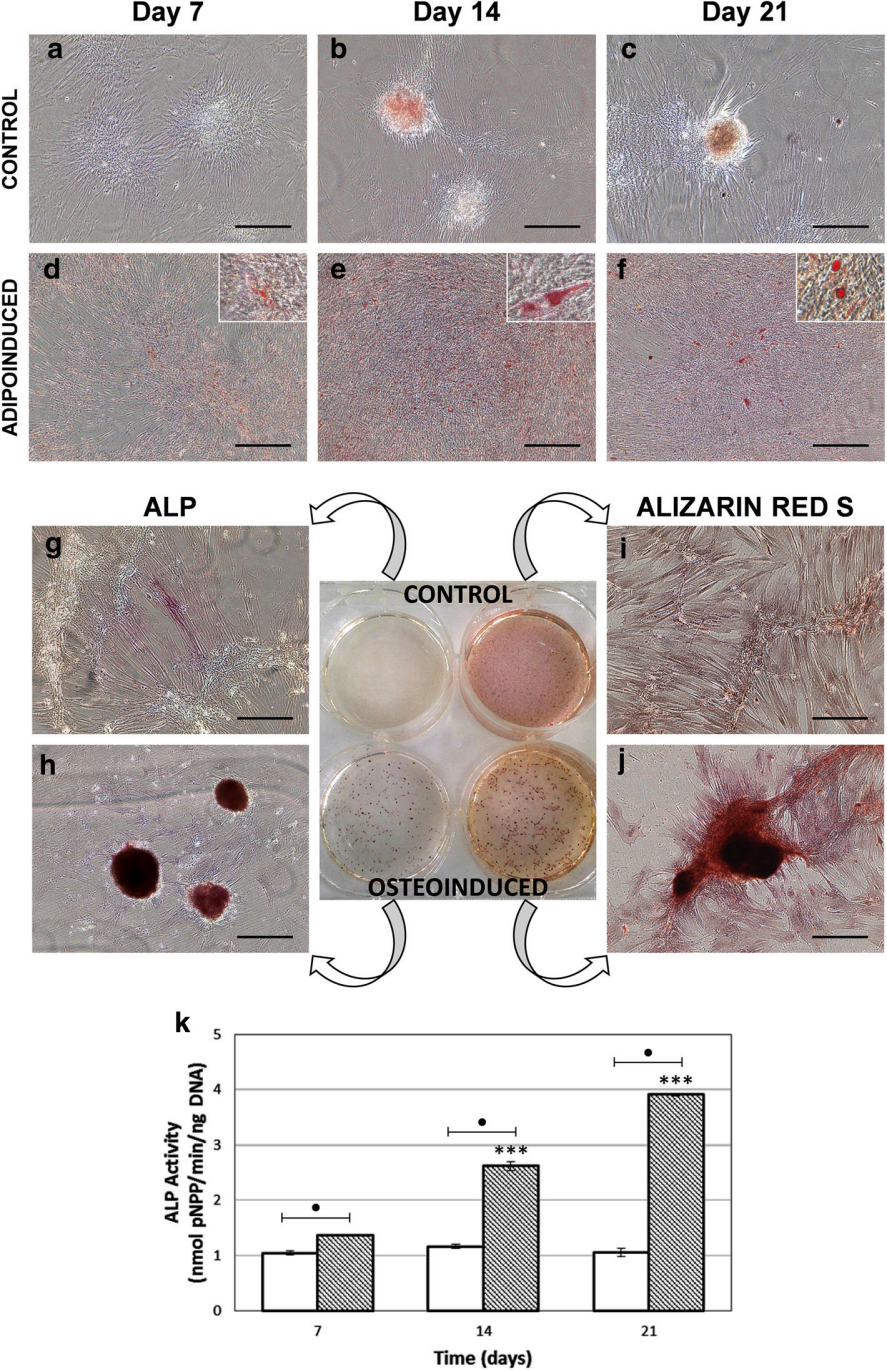
Assessment of adipogenic (**a-f**) and osteogenic (**g-k**) differentiation. Control (**a-c**) and adipo-induced (**d-f**) cultures at 7, 14 and 21 days. Positive Oil Red O staining confirmed the presence of lipid droplets only in adipogenic-induced cells. Bars, 200 μm. Inserts represent higher magnification of the specific stain. Histochemical localization of ALP (**g-h**) and Alizarin Red S (**i-j**) staining at day 21. Osteoinduced cells formed numerous nodules highly positive for ALP staining. Control cells remained Alizarin Red S negative by day 21 whereas red calcium nodules clearly appeared on the osteoinduced cultures. **k** Quantitative measurement of ALP activity at 7, 14 and 21 days. Osteoinduced cells exhibited significantly higher levels of ALP activity (*p* < 0.001) compared with the controls. Values represent the means ± SD, *n* = 3. Asterisk (*) indicates a statistically significant difference (*p* < 0.001) for the same condition at different time points, whereas (●) represents *p* < 0.001 between two groups at the same time period. Bars, 200 μm

Adipogenic differentiation was confirmed by Oil Red O staining. After culturing cells with adipogenic-inducing media for 21 days, red-stained lipid droplets were present in the cytoplasm (Fig. 4a-f).

The osteogenic differentiation potential was confirmed by histochemical localization of ALP and Alizarin Red S. In these cultures, morphological changes were observed; non-supplemented cells showed the spindle-shaped morphology, while osteoinduced cells formed numerous nodules, which were stronger stained for ALP activity (Fig. 4g-h) and Alizarin Red S (Fig. 4i-j). Consistent with these results, quantitative measurement of ALP activity showed that osteoinduced cells exhibited significantly higher levels (*p* < 0.001) compared with the controls (Fig. 4k). Moreover, a significantly calcium deposition was only detected in treated cells (*p* < 0.001) (Table 3).

**Table 3:** Quantification of calcium content as indication of mineralization

Time (days)	Treatment	Calcium (mg/dl)
21	Control	9.468 ± 0.275
	Osteoinduced	13.496 ± 0.892**

Values are expressed as the mean ± SD (*n* = 3). ** *p* < 0.01

**Fig. 5:**
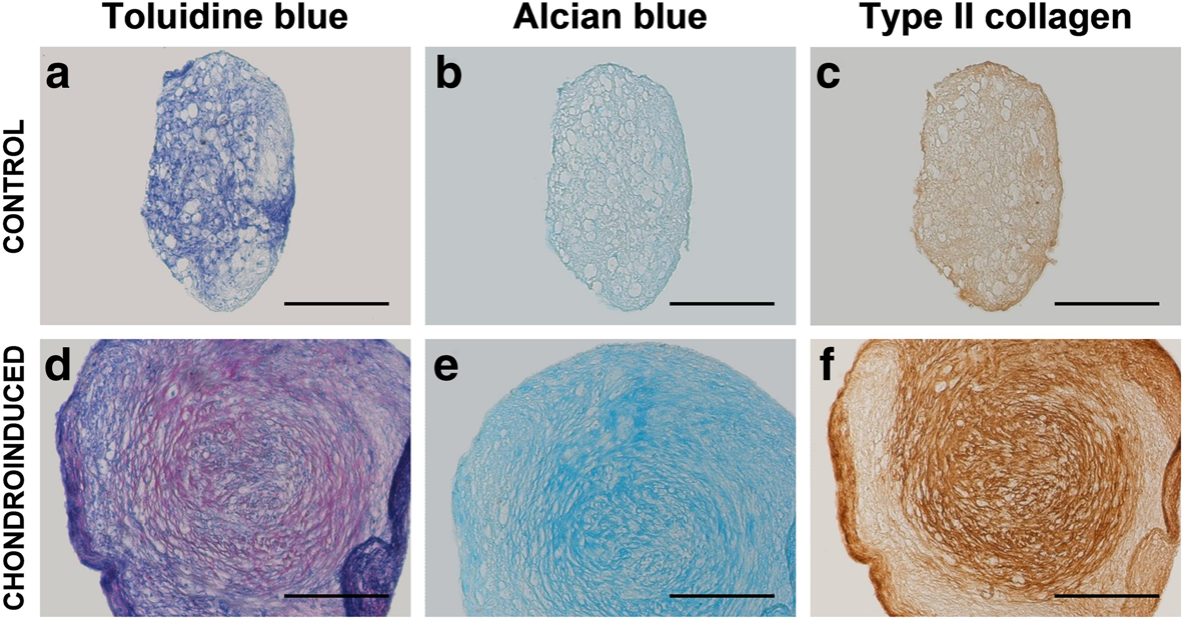
Assessment of chondrogenic differentiation. Histological sections of pellets after 21 days in the absence (**a-c**) or presence (**d-f**) of rhTGF-β1. The degree of maturation after chondrogenic differentiation was assessed by TB, AB staining and type II collagen immunohistochemistry. Pellets incubated with rhTGF-β1 clearly displayed improved chondrogenesis. Bars, 200 μm

For chondrogenic differentiation, a 3D pellet culture model was used. Histological study showed that fAd-MSCs were able to differentiate into chondrocytes. After 21 days of culture, pellets incubated with rhTGF-β1 showed characteristic chondrogenic phenotype with lacunae formation. Besides, their extracellular matrix exhibited metachromasia when stained with TB and high affinity for AB, indicating accumulation of glycosaminoglycans and proteoglycans (Fig. 5). Immunohistochemical staining revealed that pellets stimulated with rhTGF-β1 produced a deposition of cartilage-specific type II collagen.

### Karyotype

fAd-MSCs had a normal metaphase spread and karyotype.

### Immunomodulatory potential

**Fig. 6:**
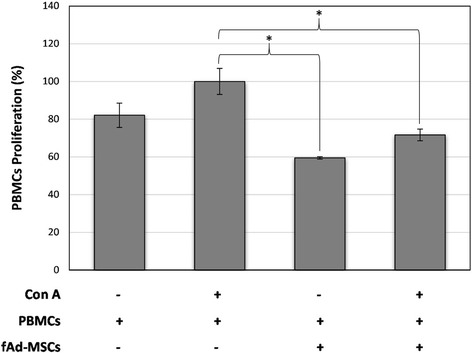
Suppression of peripheral blood mononuclear cells (PBMCs) proliferation. PBMCs were stimulated with concanavalin A (Con A) and then incubated with fAd-MSCs. Values represents the means ± SD, n = 3. Asterisk (*) indicates a statistically significant difference (*p* < 0.05)

The PBMCs proliferation was assessed by flow cytometry of CMFDA stained cells and their growth was compared to the presence or absence of fAd-MSCs at 72 h post co-culture. As depicted in Fig. 6, fAd-MSCs significantly reduced PBMCs proliferation (*p* < 0.05) in direct contact.

### Cell transplantation

**Fig. 7:**
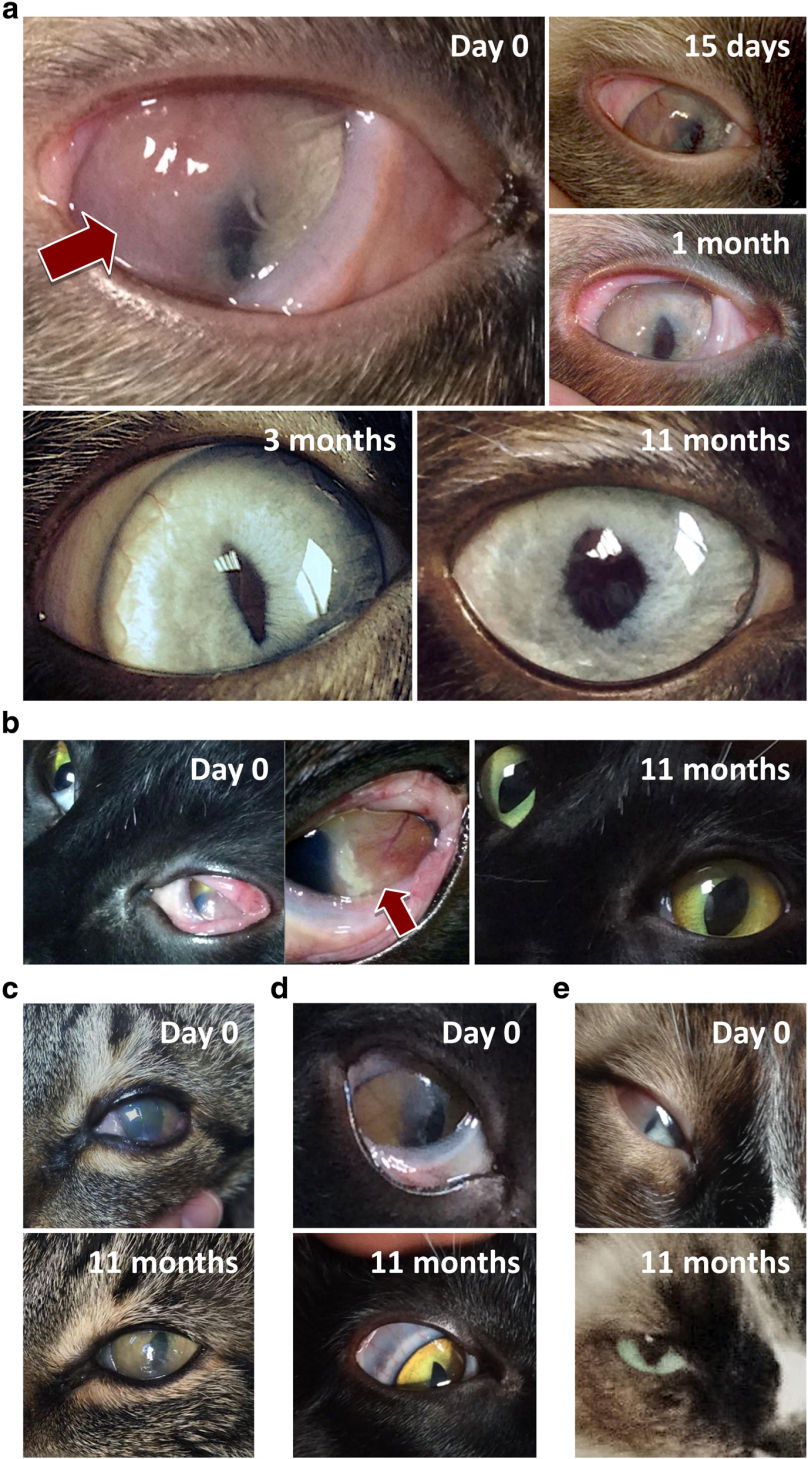
Follow-up eye images in five cats (**a-e**) with chronic eosinophilic keratitis. Animals showed a complete remission of clinical signs at 6 months and this recovery remained stable until the last follow-up at 11 months

After the first implantation, 2 of the 5 cases (Fig. 7a-b) presented decrease of ocular signs before the second implantation. Their clinical evaluation showed a significant improvement during the 4 first weeks after cell transplantation with progressive disappearance of ocular signs, beginning with a decrease of corneal plaque and a subsequent decrease in the corneal vascularization and hyperemia (Fig. 7a). In these animals, the corneal cytology was negative for eosinophils and mast cells from the 2-month follow-up. Rest of animals showed a complete remission of clinical signs and cytology at 6 months. This recovery remained stable until the last follow-up where the cornea was totally transparent with complete regression of corneal plaque, blood vessels and hyperemia, and did not show signs of worsening without topic treatment (Fig. 7).

There were no local or systemic complications during all follow-ups, without any alteration of the blood cell count and serum biochemistry profile, with respect to initial values.

## Discussion

This is the first clinical study in FEK that evaluates results after subconjunctival implantation of allogeneic fAd-MSCs with 11-months follow-up. FEK is a chronic, progressive, inflammatory disease of the cornea and conjunctiva. Its manifestation is through superficial vascularization with formation of gritty yellow-white plaques and stromal infiltration with edema of the cornea [1, 10, 29]. The disease seems to have no age, breed or sex predilection, and appears unilaterally 80% of the time [6, 29]. If left untreated, it may eventually become a bilateral condition, as being a progression of the disease [1, 10]. The characteristics and clinical signs described in our cases are consistent with published results about this disease [1, 6].

The etiology of the disease is still undetermined, but appears to be an immune response to an unknown antigenic stimulus [30], that triggers inflammation with a prominent role in the development of the symptoms and signs.

In this study, the cytology results of the affected areas showed eosinophils and mast cells in all cases (100%), similar to other studies [2, 6]. These cells are not found on healthy feline corneas, and their presence has been assumed to be pathognomonic of FEK. It is described a correlation between FHV-1 and FEK, where the FHV-1 is the most frequent cause of conjunctivitis and keratitis in domestic cats [31].

The current treatment for this disease consists of topical anti-inflammatory agent (corticosteroid) or drugs with glucocorticoid-like activity (megestrol acetate) and/or immunosuppressant (cyclosporine A) [2, 6, 11]. These agents are associated with deleterious and undesirable side effects that limit the long-term use of these drugs [26]. Therefore, corticosteroids are a poor choice in corneal diseases with FHV-1 infection, because to induce virus reactivation, potentiate corneal penetration of virus, and increase susceptibility of keratocytes to viral infection [7, 32]. On the other hand, topical cyclosporine in cats has been related with certainly grade of intolerance and adverse effects like irritation, chemosis, conjunctival hyperemia and blepharitis [1, 6].

The refractory cases of eosinophilic keratitis have been treated with megestrol acetate, however the condition can only be control but not cured, since after the cessation of treatment, recurrence of clinical signs is common. [6, 10] Also different side effects such as neoplasia, diabetes mellitus, adrenocortical suppression, behavioral changes and mammary hyperplasia have been associated with its use. [33] All these contraindications entail the need to find a safe, simple and effective treatment.

Due to the chronic disease character, the condition can only be controlled but not cured. In addition, recurrences of clinical signs are common after treatment cessation [6, 10]. All this leads to the need to find a safe, simple and effective treatment.

Feline Ad-MSCs are multipotent stem cells with capacity to differentiate, with important secretory faculty of different bioactive molecules with trophic, paracrine, anti-inflammatory and immunomodulatory functions [13, 34, 35]. Ad-MSCs have been isolated and characterized from several domestic species and are currently being used as therapeutics for a number of clinical applications in veterinary medicine [23, 24, 26, 36–39].

In our study, we used adipose tissue because it is an easily affordable and plentiful source for obtaining MSCs with a high proliferation capacity [26, 40]. Very few studies have investigated the isolation and characterization of feline MSCs [21, 23, 24, 40, 41]. Feline Ad-MSCs obtained from our donor have shown consistency in their isolation, expansion, high ratio proliferation, plastic adherent, and behavior in vitro, exhibiting its ability of adipogenic, osteogenic, and chondrogenic differentiation similar to those described for this specie [40–43].

Our FACS data are consistent with other studies, although the immunophenotype of the feline cells involves difficulty because of the limited availability of antibodies, which cross-react with this species. The expression profile corresponding with those is widely described in the literature for MSCs, as shown by our results: positive expression of CD29, CD44, CD73, CD90, STRO-1 and MHC-I, and lack of expression of hematopoietic markers CD34, CD45 and MHC-II [21, 23]. There is controversy in the literature about the use of STRO-1 as a good MSCs marker; however, some authors recommend this profile, among others, as the most useful markers for the optimal identification of MSCs [44]. The positive population has shown ability to differentiate into multiple mesenchymal lineages, such as chondroblasts, adipoblasts, osteoblasts in addition to hematopoiesis supportive cells with a vascular smooth muscle-like phenotype [45].

Furthermore, fAd-MSCs have shown in vitro both their capacity to inhibit mitogen-stimulated PMBC proliferation as well as the genomic stability of their karyotype. These results were consistent with others recently published [46, 47].

The effective use of allogeneic MSCs in patients is a new reality possible due to their low immunogenicity by lack of MHC-II [13]. This allows a rapid initiation of therapy without the need for harvesting MSCs from each patient, screening the best donors, preventing transmission of infectious diseases, and evaluating in vitro their MSC profile and immunosuppressive features [26, 46].

All animals were transplanted with cells in passage 2, allowing to obtain the necessary amount of fAd-MSCs and to avoid unnecessary additional subculturing, which is related with multipotential and proliferation rate decays, senescence, cell size increases, and chromosomal instabilities [46, 48].

Our study displays a substantial clinical improvement of ocular signs in all animals, starting with a regression of corneal proliferation and progressive decrease of neovascularization in the first weeks of treatment. This recovery remained stable until the last follow-up and showed no signs of regression or worsening. In all animals, the corneal cytology was negative for eosinophils and mast cells at the end of the follow up. None of the animals presented systemic or local complications during the study, as it has been documented for long-term studies in domestic species, which shown no adverse effects with the administration of MSCs in a large number of animals [26]. Acute phase markers were not included in the study, as no specific parameter is described for this disease [49–51].

We believe that the possible mechanism of fAd-MSCs in FEK is based on their immunomodulatory and anti-inflammatory capacities. These effects could be mediated by direct cell-cell contact and/or secretion of various soluble substances of their secretome (indolamine 2,3-dioxygenase, prostaglandin E2, TGF-β, hepatocyte growth factor, nitrous oxide, IL-10, interleukin receptor antagonist 1, etc.), as it has been recently proposed [13, 18, 47, 52].

It has been shown that MSCs, despite being good activators of angiogenesis and secreting vascular endothelial growth factor (VEGF) [53, 54], have an opposite effect on corneal angiogenesis. This fact seems to be related to the increase in the expression of thrombospondin-1 (TSP-1), which is a potent anti-angiogenic factor, and the reduction of proangiogenic factor MMP-2 that is related to inflammation [55, 56].

Regarding the humoral immunity (Th2), MSCs modulate the functions of B-lymphocytes by suppressing cell differentiation and immunoglobulin production by plasma cells. Its immunomodulatory capacity is also complemented with its significant potential to promote the generation and maintenance of the activity of different types of regulatory T cells [13, 18, 52].

Our results are quite encouraging considering that we have treated animals with FEK refractory to current conventional treatments, where there was no viable alternative to solve their pathology. We demonstrated that fAd-MSCs subconjunctival implantation is a novel, safe, effective and sustained strategy for FEK refractory to current treatments, with a clinical significant improvement that allows removing much of the medication that must be applied, improving the economic cost and quality of life of animals.

The cat has been employed as a model for cellular therapy with MSCs in various pathologies [21, 23, 24]. The characterization of adipose tissue-derived MSCs has demonstrated to be similar in their properties and behavior to human MSCs [47, 56]. Therefore, our study can be used as an immunomodulation model for Th2-mediated response.

In spite of the limitations of this study, lack of control group and few treated animals, we believe that the results are relevant.

## Conclusions

This is the first study to demonstrate the efficacy of fAd-MSCs in FEK. Allogeneic fAd-MSCs subconjunctival implantation is a safe, effective, and relatively simple therapy for FEK in cats, with a significant improvement of corneal surface signs associated with the disease during at least 11 months.

## Abbreviations

7-AAD: 7-aminoactinomycin D
AB: Alcian blue
ALP: Alkaline phosphatase
BSA: Bovine serum albumin
DMEM: Dulbecco’s modified Eagle’s medium
FACS: Fluorescence-activated cell sorting
fAd-MSCs: Feline adipose-derived mesenchymal stromal cells
FBS: Fetal bovine serum
FEK: Feline eosinophilic keratitis
FFV: Feline foamy virus
FHV-1: Feline herpes virus-1
FPK: Feline proliferative keratitis
IBMX: 3-isobutyl-1-methylxanthine
IL: Interleukin
MHC: Major histocompatibility complex
PBMCs: Peripheral blood mononuclear cells
PCR: Polymerase chain reaction
STT-1: Schirmer tear test
TB: Toluidine blue
TBSS: Tyrode’s balanced salt solution
TGF-β: Transforming growth factor β
